# Microwave-Assisted Rapid Synthesis of Eu(OH)_3_/RGO Nanocomposites and Enhancement of Their Antibacterial Activity against *Escherichia coli*

**DOI:** 10.3390/ma15010043

**Published:** 2021-12-22

**Authors:** Kun-Yauh Shih, Shiou-Ching Yu

**Affiliations:** Department of Applied Chemistry, National Pingtung University, Pingtung County 90003, Taiwan; sunny1030915@gmail.com

**Keywords:** antibacterial, graphene oxide, europium(III) hydroxide, microwave-assisted synthesis, nanocomposites

## Abstract

Nanomaterials with high antibacterial activity and low cytotoxicity have attracted extensive attention from scientists. In this study, europium (III) hydroxide (Eu(OH)_3_)/reduced graphene oxide (RGO) nanocomposites were synthesized using a rapid, one-step method, and their antibacterial activity against *Escherichia coli* (*E. coli*) was investigated using the synergistic effect of the antibacterial activity between Eu and graphene oxide (GO). The Eu(OH)_3_/RGO nanocomposites were prepared using a microwave-assisted synthesis method and characterized using X-ray diffraction, scanning electron microscopy, photoluminescence spectroscopy, Raman spectroscopy, and Fourier-transform infrared spectroscopy. Raman sprectroscopy and X-ray diffraction confirmed the pure hexagonal phase structure of the nanocomposites. Further, the antibacterial properties of Eu(OH)_3_/RGO were investigated using the minimum inhibitory concentration assay, colony counting method, inhibition zone diameter, and optical density measurements. The results revealed that the Eu(OH)_3_/RGO exhibited a superior inhibition effect against *E. coli* and a larger inhibition zone diameter compared to RGO and Eu(OH)_3_. Further, the reusability test revealed that Eu(OH)_3_/RGO nanocomposite retained above 98% of its bacterial inhibition effect after seven consecutive applications. The high antibacterial activity of the Eu(OH)_3_/RGO nanocomposite could be attributed to the release of Eu^3+^ ions from the nanocomposite and the sharp edge of RGO. These results indicated the potential bactericidal applications of the Eu(OH)_3_/RGO nanocomposite.

## 1. Introduction

The growth and spread of microorganisms on the surface of public infrastructure directly affect human life [1,2]. Particularly, owing to the increase in the development of resistance by various bacteria species to existing antibiotics, the clinical management/control of bacterial infections has emerged as a medical challenge for humans [3]. Traditionally, bacteria are controlled using conventional bactericides. However, the negative effects of some bactericides on the environment and on humans have restricted their further application. In addition, the increase in the development of resistance by various bacteria species to conventional bactericides has limited the further application of these bactericides [4]. To address these issues, the use of antimicrobial agents has emerged as a promising approach. Antimicrobial agents can be divided into two categories: organic and inorganic antimicrobial agents. Organic antibacterial agents mainly include organic acids, alcohols, esters, and phenols and have attracted widespread attention owing to their low cost and good bactericidal effect. In addition, these agents can interact with the combination of ions between the cell wall and the membrane of microorganisms, which results in the denaturing of the proteins of the microorganism, thus damaging them [5]. However, compared to inorganic materials, organic antibacterial agents are generally less stable at higher temperatures. Consequently, this hinders the design of stable antibacterial materials that can withstand harsh processing conditions [6]. To overcome these problems, inorganic nanomaterials have emerged as promising antimicrobial materials. Inorganic antimicrobial materials have attracted increasing attention owing to their resistance to acids and alkali, antibacterial durability, washing resistance, biocompatibility, non-toxicity, and lack of secondary pollution; however, their susceptibility to oxidation and discoloration has restricted their effective application [7,8]. Therefore, it is essential to develop new antibacterial agents through environmentally friendly and cost-effective technology [9,10,11]. Nevertheless, as a safe and green material, antibacterial materials have attracted increasing attention, and the importance of inorganic reagents is increasing. Currently, the development of new antimicrobial materials and antimicrobial strategies has attracted tremendous research attention. Recently, nanomaterials have attracted considerable attention as effective antibacterial agents [6,12,13]. This could be attributed to the high surface-to-volume ratio of nanoparticles and the presence of a high number of contact points for bacteria, which enhances the antimicrobial efficiency of nanomaterials and reduces the processing cost compared to inorganic metal materials [12,14,15,16,17,18].

Rare earth elements (REE) exhibit excellent optical, electrical, and magnetic properties due to their special 4f electron configuration and have been applied in permanent magnetic materials, catalysts, and antibacterial materials [19,20]. In addition, REE have attracted increasing attention owing to their low cost, large surface area, good colloidal behavior, and low cytotoxicity. The addition of REE to antimicrobial materials may enhance their antibacterial performance. A previous study demonstrated the non-toxic nature of europium (III) hydroxide (Eu(OH)_3_) nanorods for in vitro and in vivo systems [21]; therefore, Eu(OH)_3_ was employed as an antimicrobial material in this study. Although Eu(OH)_3_ nanoparticles kill bacteria by destroying bacterial cell membranes, the aggregation of Eu in solutions leads to a reduction in the specific surface area of Eu(OH)_3_ nanoparticles in solution, thus significantly reducing their bactericidal activity.

Graphene has been used extensively in recent years for material research [22]. Particularly, graphene oxide (GO) has attracted attention owing to its easy processibility, low cost, and environmental friendliness compared to other carbon materials. In addition, the modification of GO using a chemical reaction to prepare hydroxyl (OH) and carboxyl groups containing graphene has been explored. These OH and carboxyl groups containing graphene exhibit a good water solubility and a large surface area, which enhance the contact between the material and other substances, thus facilitating the reaction. In addition, GO exhibits specific interactions with microorganisms and induces antimicrobial action via cell membrane damage and oxidative stress [23]. To address the aggregation of nanoscale particles and the non-uniform distribution during membrane formation, GO nanosheets are used as a platform to enhance their dispersibility. This is achieved via the synergistic effects between the hydrophilic GO layer and nanoparticles. In addition, the carboxyl and OH functional groups of GO are essential for the formation of hybridized nanostructures with various nanoparticles [24,25].

Various methods, such as ultrasonication [26,27], hydrothermal method [28], thermal decomposition method [29], and the sol-gel method [30], have been employed for the preparation of nanocomposite antimicrobial materials, which exhibited good antimicrobial activities. However, the high energy consumption of these methods owing to longer heating times, the longer reaction times owing to multiple iterative steps, and the high chemical consumption for various solvents and precursors have limited their further application. To overcome these challenges, in this study, we synthesized antimicrobial nanocomposites using a simple, efficient, time-saving, and environmentally friendly one-step method. Previous studies employed microwave-assisted synthesis for the synthesis of nanomaterials owing to its attractive properties, such as short reaction time, narrow size distribution, and high purity [31,32]. In addition, previous studies successfully prepared antimicrobial materials with excellent antimicrobial ability, such as AgGO [33], Ag/rGO [34], CdO–CuO [35], and CdO–ZnO–MgO [36], using the microwave-assisted synthesis method.

In this study, we prepared Eu(OH)_3_/RGO nanocomposite as a new antibacterial nanocomposite using the microwave-assisted method. This is an environmentally friendly method that utilizes microwave energy in chemical reactions, thus ensuring that the reaction is faster, cleaner, and cheaper compared to traditional methods [37]. The antibacterial activity of the Eu(OH)_3_/RGO nanocomposite against *Escherichia coli*, a Gram-negative bacterium, was investigated, and the results revealed that the Eu(OH)_3_/RGO nanocomposite effectively inhibited the growth of *E. coli*.

## 2. Materials and Methods

### 2.1. Materials Used

The chemicals used in this study included natural graphite powder (Alfa Aesar, Ward Hill, MA, USA, 99.99%), europium(III) nitrate pentahydrate (Sigma–Aldrich, St. Louis, MO, USA, 99.9%), potassium hydroxide (Nihon Shiyaku reagent, Tokyo, Japan, 95%), sulfuric acid (H_2_SO_4_; Nihon Shiyaku reagent, 99.99%), acetone (Nihon Shiyaku reagent, 95%), sodium nitrate (Hayashi Pure Chemical Ind, 99.5%), hydrogen peroxide (Showa chemical, Tokyo, Japan, 30%), Silver powder 30 nm (UniRegion Bio-Tech, 99.9%), Luria–Bertani (LB) broth (UniRegion Bio–Tech, Taipei, Taiwan), and Mueller–Hinton agar 2 (Sigma–Aldrich). GO was prepared via the modified Hummers method. The purity of the chemicals used in this study was of analytical grade and the chemicals were used as received without any treatment. All solutions were prepared using deionized water in the experiment.

### 2.2. Synthesis of the Eu(OH)_3_/RGO Nanocomposites

**Figure 1:**
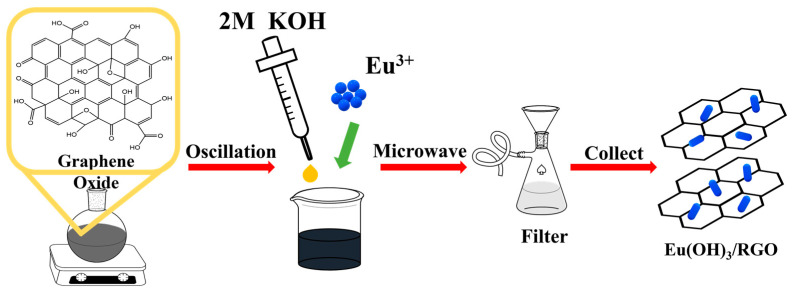
Schematic illustration of the synthesis process of the europium hydroxide (Eu(OH)_3_)/reduced graphene oxide (RGO) nanomaterial. KOH: Potassium hydroxide.

First, GO was synthesized from natural graphite powder using H_2_SO_4_ and KMnO_4_ as oxidizing agents according to a modified Hummers method and our previous study [38,39]. Subsequently, the prepared GO was washed with deionized water to remove residual salts and acids, after which the GO was dried in an oven at 70 °C. Eu(OH)_3_/RGO nanocomposites were prepared under microwave irradiation. Briefly, 50 mg of GO were dispersed in 30 mL deionized water using ultrasonication at room temperature. Thereafter, 0.56 mmol of Eu(NO_3_)_3_ and 5 mL of 2 M KOH were added to the ultrasonicated solution, after which the mixture was subjected to ultrasonication for 30 min. Subsequently, the mixture was moved to a Teflon-lined vessel and subjected to microwave heating at 130, 160, 190, and 220 °C for 10 min in a Flexiwave T660 Microwave (Milestone) to obtain Eu(OH)_3_/RGO samples. Lastly, the as-prepared Eu(OH)_3_/RGO samples were filtrated and dried in an oven at 75 °C for 12 h. For comparison, pure Eu(OH)_3_ was prepared using a similar process. The Eu(OH)_3_/RGO nanocomposites microwaved at 130,160, 190, and 220 °C for 10 min were labeled as Eu(OH)_3_/RGO 130, Eu(OH)_3_/RGO 160, Eu(OH)_3_/RGO 190, and Eu(OH)_3_/RGO 220, respectively. The Eu(OH)_3_ microwaved at 220 °C for 10 min was labeled as Eu(OH)_3_ 220. The schematic illustration of the synthesis process is shown in Figure 1.

### 2.3. Characterization

The crystalline structure and phase purity of the samples were characterized using X-ray powder diffraction (XRD, Mutiflex MF2100, Rigaku Co., Ltd., Tokyo, Japan) with the CuKα radiation (1.5418 Å). The surface morphology of the samples was characterized using scanning electron microscopy (SEM, Joel-JSM 6390) at an acceleration voltage of 15 kV. Raman spectra of the samples were obtained using a Princeton Instruments Acton SP2500 monochromator/spectrograph equipped with a Spec-10 system with a nitrogen-cooled CCD detector. In addition, Spectra-Physics Beamlock 2080 Krypton laser with a 647.1-nm line was used as the excitation source for the Raman signal. The photoluminescence of the samples was measured using a Hitachi F-7000 spectrometer with a Xe Lamp light source. The surface functionalization and chemical bonding properties of the nanocomposites were analyzed using Fourier transform infrared (FT–IR) spectroscopy (Technologies Cary 630, Cary, NC, USA) in the wavelength ranges from 4000 to 500 cm^−1^.

### 2.4. Antibacterial Ability of Eu(OH)_3_/RGO

#### 2.4.1. Culture Medium and Culture Conditions

The Freeze-dried *E. coli* was activated twice in BHI broth and incubated at 37 °C for 18 h. Then, a loop of bacterial suspension was inoculated to LB at 37 °C for 18 h. To obtain isolated colonies, the suspension was streaked in Petri dishes containing count agar plates and incubated at 37 °C for 20 h.

#### 2.4.2. Minimum Inhibitory Concentration (MIC)

In this study, MIC was defined as the lowest concentration of the sample required to inhibit bacteria growth after incubation. In this study, the MIC was determined using the broth microdilution method. The stationary phase *E. coli* strain used for this investigation was cultivated in a Mueller–Hinton broth incubated in 96-well plates at 37 °C for 24 h. The *E. coli* suspension (10^4^ CFU/mL) was treated with different concentrations of Eu(OH)_3_/RGO (1, 2, 4, 8, 16, 32, 64, 128, 256, 512, 1024, and 2048 µg/mL) and Eu(OH)_3_ (1, 2, 4, 8, 16, 32, 64, 128, 256, 512, 1024, and 2048 µg/mL), and the MIC of the samples was observed after 24 h.

#### 2.4.3. Colony Counting Method

The antibacterial ability of the samples was investigated using the flat colony counting method. A sample concentration of 8 µg/mL was immersed in 100 mL *E. coli* suspension (10^4^ CFU/mL) for 30 min. To obtain the most accurate experimental results, Eu(OH)_3_/RGO nanocomposites were completely and uniformly dissolved in the suspensions. An aliquot (0.15 mL) of the aforementioned suspension was spread onto a Muller–Hinton agar plate and the number of colonies was counted after 24 h. All experiments were independently repeated three times to calculate the mean and standard deviation. The number of colonies on the plates was counted using the standard plate count technique (CFU) [40].

The cell viability was calculated using the following equation [41].

(1) $$E=\frac{b}{a}\times \;100\%$$

where *a* is the count of Blank and *b* is the count of living cells after immersing in the sample.

#### 2.4.4. Optical Density (O.D.)

The optical density (O.D.) of the samples was measured using the shaking flask method, and the obtained values were plotted against the *E. coli* cultured time. To measure the O.D., first, the 100 mL as-prepared bacterial suspension (10^4^ CFU/mL) was immersed in different concentrations of Eu(OH_)3_/RGO 220 (1, 4, 8, 16, 32 µg/mL) separately in an Erlenmeyer flask. Subsequently, the Erlenmeyer flask was incubated at 37 °C in a shaking incubator. To compare the bacterial growth curves between three different materials, we prepared 0.8 mg (8 µg/mL) of Eu(OH_)3_/RGO, Eu(OH)_3_, and RGO and measured the optical density every 3 h for 24 h. The growth of *E. coli* was detected by measuring the O.D. of the solution using a CT-2200 UV/Vis spectrophotometer (ChromTech Co., Ltd., Apple Valley, MN, USA) spectrophotometer at 600 nm every 3 h. The change in the turbidity at each concentration was calculated by subtracting the turbidity of the corresponding blank from the turbidity of the Erlenmeyer flask containing the bacteria and antibacterial agent.

#### 2.4.5. Inhibition Zone

The inhibition zone was determined using the disk diffusion method. Briefly, the Nutrient agar was poured onto a Petri dish and left overnight at room temperature to solidify. Subsequently, 150 µL of *E. coli* solution (10^4^ CFU/mL) was spread on the plate. Thereafter, 0.1 mg/mL of the sample and 0.1 mg/mL of ampicillin, which was used as the control group, were prepared. Thereafter, 250 µL of the sample were placed in a well carefully made from a stainless-steel straw. Lastly, the Petri dishes were cultured at 37 °C for 18 h. The inhibition zones were observed and measured using a Vernier caliper. The experiment was repeated three times and the mean value and standard deviation were recorded.

#### 2.4.6. Reutilization Experiment

To verify the cyclic antibacterial performance of the Eu(OH)_3_/RGO 220 nanocomposite, the nanocomposite was collected by centrifuge after each assay for a total of seven cycles. Next, 12 µg of Eu(OH)_3_/RGO 220 nanocomposite were mixed with 1.5 mL of *E. coli* suspension (10^4^ CFU/mL) and incubated in a shaking incubator at 37 °C and 120 rpm for 30 min. Subsequently, the Eu(OH)_3_/RGO 220 nanocomposites were cleaned with deionized water and ethanol and dried for 1 h in an oven at 50 °C for the next round of colony counting. The process was repeated seven times.

#### 2.4.7. Statistical Analysis

For statistical analysis of data, multiple comparisons were performed using one-way analysis of variance (ANOVA) followed by the LSD test for post hoc analysis. Data were analyzed using IBM SPSS statistics 22. Data with a *p*-value < 0.05 were considered to be statistically significant. The significance is indicated as * *p* < 0.05 and ** *p* < 0.01 in the figures and table.

## 3. Results and Discussion

### 3.1. Characterization of Eu(OH)_3_/RGO

#### 3.1.1. XRD Analysis

**Figure 2:**
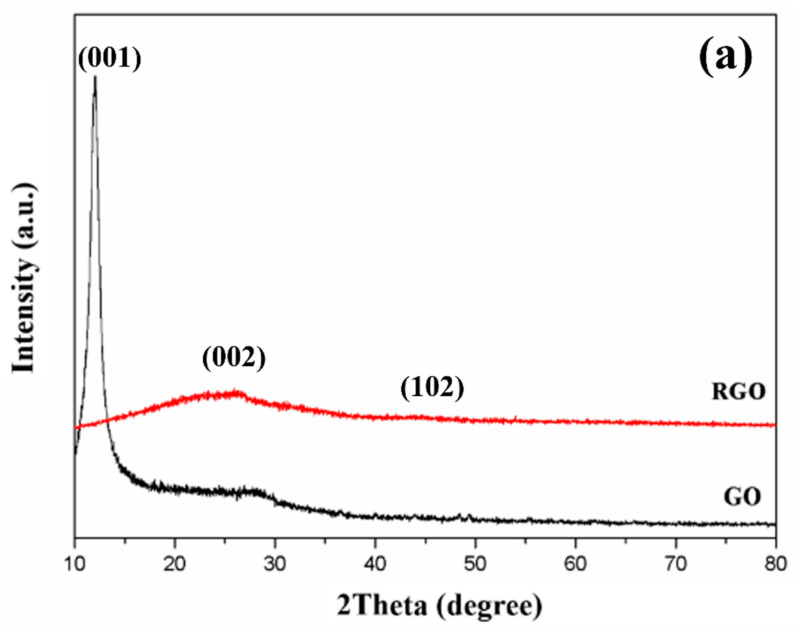

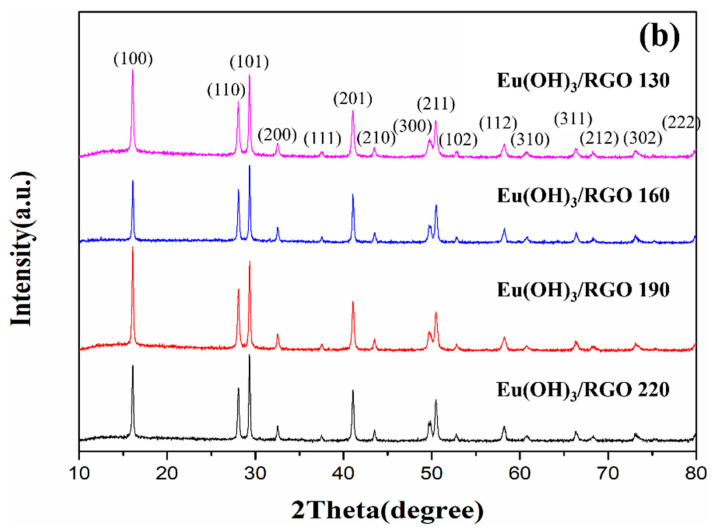
X-ray diffraction (XRD) patterns of (**a**) GO, RGO and (**b**) Eu(OH)_3_/RGO nanocomposites.

The successful formation of the nanocomposites was verified by investigating the phase purity and crystalline structure of GO and Eu(OH)_3_/RGO using XRD. Figure 2a shows the XRD patterns of GO and RGO. A notable peak was observed at 2θ = 11.7° in the XRD pattern of GO, which could be attributed to the (001) crystalline plane of GO, indicating the successful oxidation of graphite. In addition, the broad peak observed at 2θ = 21° and the disappearance of the peak at 2θ = 11.7° in the XRD pattern of RGO confirmed the successful reduction of GO [42].

Figure 2b shows the XRD pattern of Eu(OH)_3_/RGO nanocomposites prepared at various temperatures. Due to the XRD pattern of RGO not being obvious, the XRD patterns of Eu(OH)_3_/RGO nanocomposites can be ascribed to pure hexagonal phase (space group P63/m) Eu(OH)_3_ ( JCPDS 83-2305: a = b = 6.35°A, c = 3.65°A ) [43]. The major peaks in the XRD patterns of the Eu(OH)_3_/RGO nanocomposites were observed at 2θ = 16.1°, 28.1°, 29.3°, 32.5°, 37.4°, 41.0°, 43.4°, 49.8°, 50.4°, 52.7°, and 58.2°, corresponding to the (100), (110), (101), (200), (111), (201),(210), (300), (211), (102), (112), (310), (311), (212), and (302) crystal planes, respectively [43]. In addition, the XRD patterns exhibited sharp reflections at small angles and wide reflections at high angles, indicating the layered structure of the nanocomposites [44]. Furthermore, no diffraction peaks corresponding to the presence of other impurities were detected. In addition, the typical diffraction peaks of RGO (002) were not observed, indicating that the crystal growth of Eu(OH)_3_ between the interlayer of the RGO sheet resulted in the exfoliation of RGO [45]. The results confirmed that the Eu(OH)_3_ nanoparticles were loaded on the surface of RGO, indicating the successful synthesis of the nanocomposites.

#### 3.1.2. Raman Spectroscopy

**Figure 3:**
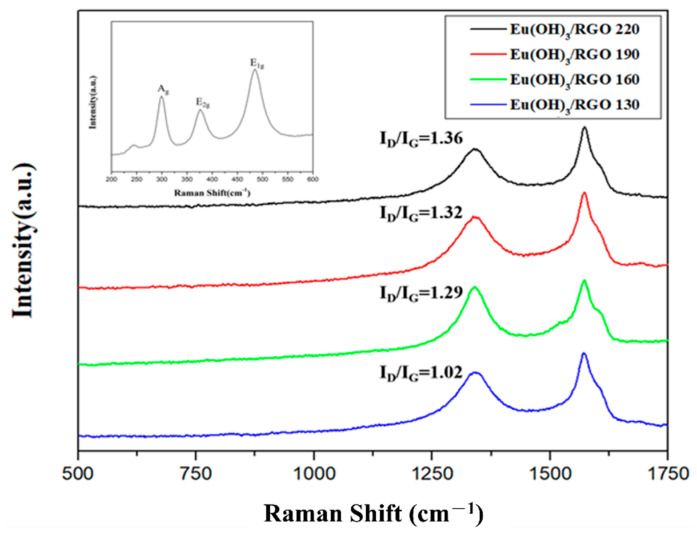
Raman spectra of the Eu(OH)_3_/RGO nanocomposites (inset shows the Raman spectrum of Eu(OH)_3_).

Raman spectroscopy is an important technique for characterizing carbon materials as it can closely examine the electron structure of carbon materials. Figure 3 shows the Raman spectra of the Eu(OH)_3_/RGO nanocomposites. Two major peaks corresponding to the D band and G band were observed at 1350 and 1580 cm^−1^, respectively. The D band corresponded to the local defects and disorder of the graphite layers, whereas the G band, which is related to the sp^2^ hybridized structure [46], corresponded to the crystallizability and symmetry of the graphitic carbonaceous materials [47]. In addition, three Raman peaks were observed at 300.9, 376.1, and 485.4 cm^−1^ in the Raman spectra of Eu(OH)_3_ with a hexagonal crystal phase (P63/m), which could be attributed to A_g_ translatory, E_2g_ translatory, and E_1g_ libration modes, respectively (Figure 3) [20]. The vibrational mode representations were expressed as ^4^A_g_ + ^3^B_g_ + ^2^E_1g_ + ^5^E_2g_ +^2^A_u_ + ^4^B_u_ + ^4^E_1u_ + ^2^E_2u_, where ^4^A_g_, ^2^E_1g__,_ and ^5^E_2g_ were active Raman peaks [43,48]. The frequencies of these bands were consistent with the XRD results. Generally, the I_D_/I_G_ value is used to determine the degree of order or disorder of carbon materials [47]. The composite degree increased with an increase in the I_D_/I_G_ value. In this study, the I_D_/I_G_ value increased gradually with an increase in the synthesis temperature of Eu(OH)_3_/RGO. The I_D_/I_G_ values of the Eu(OH)_3_/RGO 130, Eu(OH)_3_/RGO 160, Eu(OH)_3_/RGO 190, and Eu(OH)_3_/RGO 220 nanocomposites were 1.02, 1.29, 1.32, and 1.36, respectively. The highest I_D_/I_G_ value was observed at 220 °C, indicating that this is the optimum temperature for the synthesis of Eu(OH)_3_/RGO 220.

#### 3.1.3. FTIR Spectroscopy

**Figure 4:**
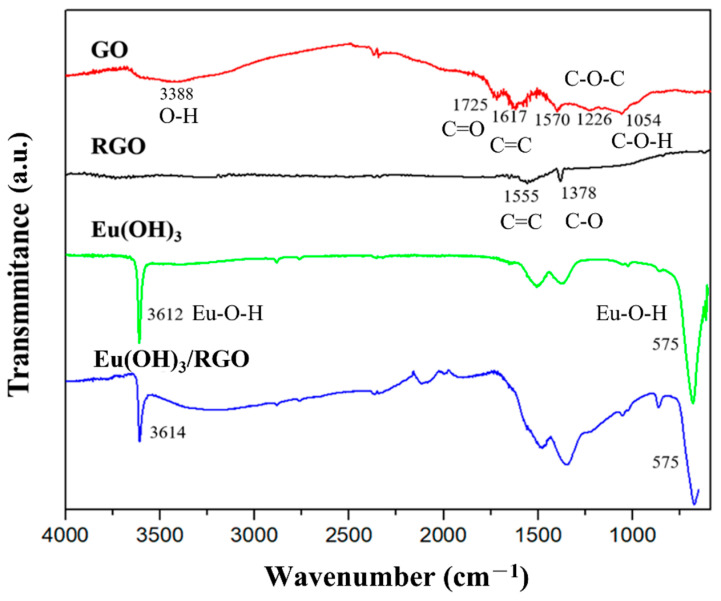
Fourier transform-infrared (FT-IR) spectra of GO, RGO, and Eu(OH)_3_ nanorods and Eu(OH)_3_/RGO 220 nanocomposites.

Figure 4 shows the FTIR spectra of pure GO, RGO, Eu(OH)_3_, and Eu(OH)_3_/RGO 220. Two peaks were observed in the spectra of the GO nanosheets at 1725 and 1617 cm^−1^, which correspond to the C=O carbonyl stretching of COOH groups and C=C stretching vibration, respectively. Two additional peaks were observed at 1054 and 1226 cm^−1^, which correspond to the carboxyl stretching of C–O–H and C–O–C vibration, respectively [49]. Furthermore, a broadband peak was observed at 3338 cm^−1^, which was ascribed to a strong stretching mode of the OH group. In contrast, the broadband peak at 3338 cm^−1^ disappeared in the FT-IR spectrum of RGO, indicating the absence of OH groups in RGO owing to the reduction of RGO from GO. In addition, the absorption peak of the oxygenous groups was not observed in the FT-IR spectrum of RGO, and two new absorption peaks were observed at 1555 and 1378 cm^−1^ [47]. In addition, the peaks at 1725, 1226, and 1054 cm^−1^ disappeared in the FT-IR spectrum of RGO, which could be attributed to the successful reduction of GO to RGO. Two peaks corresponding to Eu–O–H bending vibration and Eu–O–H stretching of Eu(OH)_3_ were observed at 575 and 3612 cm^−1^ bands in the FT-IR spectrum of Eu(OH)_3_, respectively [20]. In addition, three strong absorption bands were observed at 1300–1600, 1900–2000, and 2200–2300 cm^−1^, and two peaks were observed at 1394 and 1495 cm^−1^, which corresponded to the stretching frequency of carbonate ions absorbed from the air [50]. Furthermore, a peak was observed at 1018.45 cm^–1^, which could be attributed to the vibration of the carbonate ions, indicating the presence of carbonate ions in the compound. A similar absorption band was observed in the FT-IR spectrum of Eu(OH)_3_/RGO. Furthermore, the presence of Eu had no significant effect on the reduction process.

#### 3.1.4. Morphological Characterization

**Figure 5:**
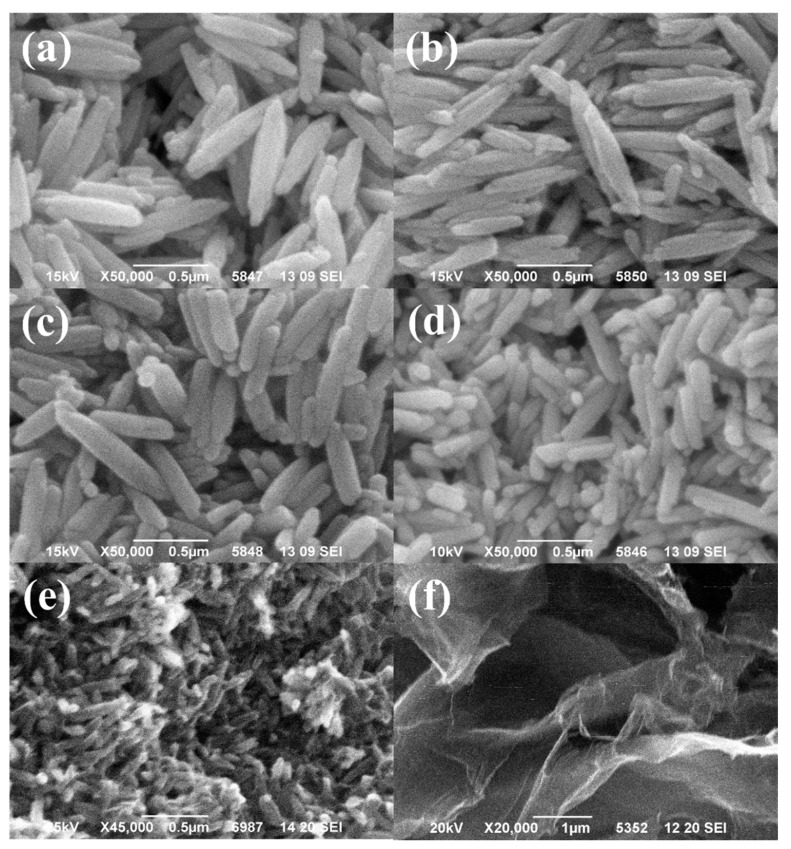
Scanning electron microscopy (SEM) image of samples. (**a**) Eu(OH)_3_/RGO 130, (**b**) Eu(OH)_3_/RGO 160, (**c**) Eu(OH)_3_/RGO 190, (**d**) Eu(OH)_3_/RGO 220, (**e**) Eu(OH)3 220, and (**f**) RGO.

The surface morphology of Eu(OH)_3_, Eu(OH)_3_/RGO nanocomposites, and GO was characterized using SEM, and the SEM images are shown in Figure 5. The SEM images revealed that RGO exhibited a flaky and slightly wrinkled surface. In addition, Eu(OH)_3_ exhibited an approximately uniform-sized particles’ rod morphology. The average rod lengths of Eu(OH)_3,_ Eu(OH)_3_/RGO 130, Eu(OH)_3_/RGO 160, Eu(OH)_3_/RGO 190, and Eu(OH)_3_/RGO 220 obtained by 100 random measurements were 600, 630, 471, 158, and 97 nm, respectively (Table 1). In addition, a uniform distribution of non-agglomerated Eu(OH)_3_ nanoparticles on the RGO sheet was observed in the SEM image of the Eu(OH)_3_/RGO nanocomposites. Furthermore, the surface of RGO in the Eu(OH)_3_/RGO nanocomposites was covered with Eu(OH)_3_ nanoparticles, verifying the good combination of Eu(OH)_3_ and RGO in the nanocomposite. In addition, the Eu(OH)_3_ nanoparticles were trapped between the RGO sheets, and the uniform embedding of Eu(OH)_3_ inhibited the restacking of the RGO layers. Simultaneously, the RGO sheets prevented the agglomeration of Eu(OH)_3_ nanoparticles and promoted the uniform distribution of Eu(OH)_3_ nanoparticles on the surface of the RGO [49]. In addition, the particle size of the nanocomposite decreased with an increase in the synthesis temperature. Accordingly, the specific surface area increased with a decrease in the particle size, which increased the area in contact with the *E. coli* suspension, thereby increasing the antibacterial ability of the nanocomposite.

#### 3.1.5. Fluorescence Properties

**Figure 6:**
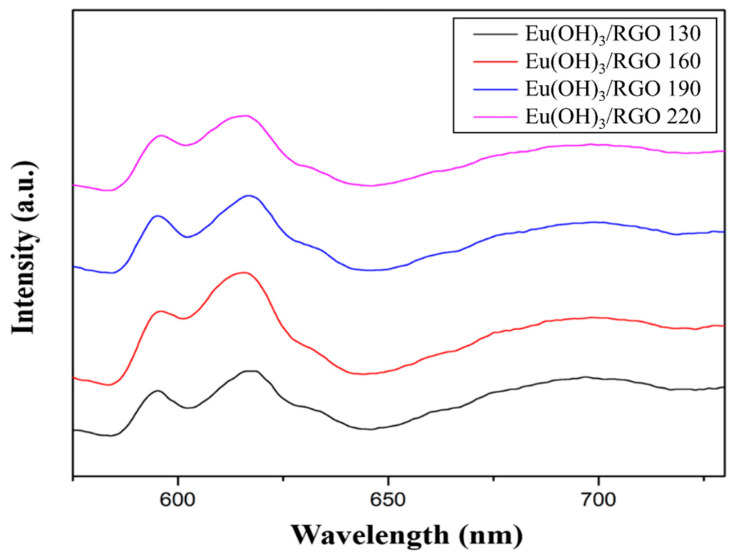
Photoluminescence spectra of Eu(OH)_3_/RGO. A wavelength (λ_ex_) of 395 nm was used for fluorescence excitation.

The emission spectra of Eu(OH)_3_/RGO nanocomposites synthesized at different temperatures obtained at a fixed excitation wavelength of 395 nm are shown in Figure 6. Despite the synthesis of the samples using a microwave-assisted method, the samples exhibited a relatively strong Eu^3+^ peak emission intensity, which could be attributed to its good morphology [51]. In addition, two peaks were detected in the fluorescence spectra of the samples between 580 and 730 nm, which were attributed to the ^5^D_0_ → ^7^F_J_ (J = 1, 2, 3, 4) transitions of the Eu (III). Furthermore, additional peaks were observed at 593, 617, 650, and 689 nm, which corresponded to J = 1, 2, 3, and 4, respectively [52]. In addition, the higher energy emissions of ^5^D_1_ and ^5^D_2_ in Eu(OH)_3_/RGO were quenched by nonradiative relaxation to ^5^D_0_ owing to the high energy OH phonons [53]. Furthermore, the emission spectrum of Eu^3+^ was very sensitive to the local symmetry of the crystal field. The peak at 593 nm corresponded to the magnetic dipole transition of ^5^D_0_ → ^7^F_1_, which had no significant effect on the symmetry of the crystal field. An additional peak corresponding to the electric dipole transition from ^5^D_0_ → ^7^F_2_ was observed at 617 nm, which could be attributed to the symmetrical torsion of Eu^3+^ in the crystal [44].

### 3.2. MIC

In this study, the MIC of the RGO, Eu(OH)_3_, and Eu(OH)_3_/RGO samples was measured using the broth microdilution method. Briefly, different concentrations of Eu(OH)_3_/RGO 220, Eu(OH)_3_ 220, and RGO were immersed in LB broth containing bacterial suspension and cultured at 37 °C for 24 h. The final number of bacteria in the MIC assay was only 1–2 single colonies found on the agar. The MICs of the Eu(OH)_3_/RGO 220, Eu(OH)_3_ 220, and RGO samples against *E. coli* were 8, 32, and 256 µg/mL, respectively (Table 2). The Eu(OH)_3_/RGO 220 nanocomposites effectively inhibited the growth of *E. coli*. These results indicated that the antibacterial activity of Eu(OH)_3_/RGO nanocomposites was higher than those of Eu(OH)_3_ and RGO.

### 3.3. Optical Density

**Figure 7:**
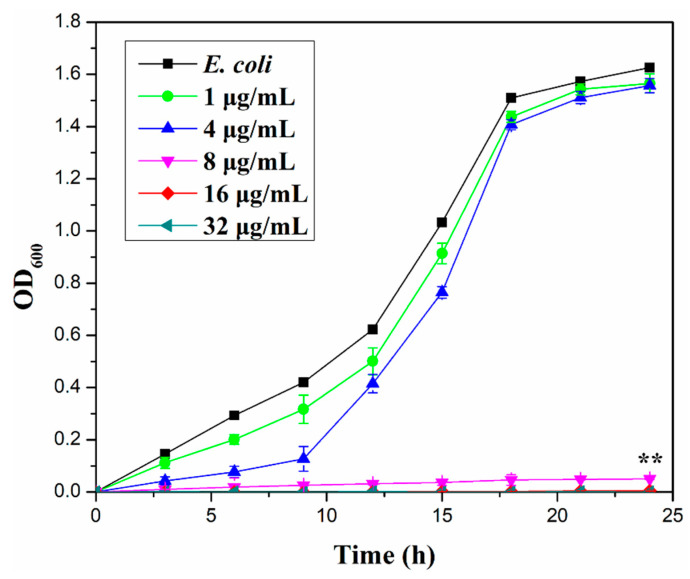
Growth curves of 100 mL *E. coli* suspension (10^4^ CFU/mL) exposed to 0.1, 0.4, 0.8, 1.6, and 3.2 mg of Eu(OH)_3_/RGO after 24 h. Error bars represent the standard deviations (n = 3; n = numbers of experimental results); ** *p* < 0.01 indicates growth curve at 24 h was statistically significant compared with the control group.

**Figure 8:**
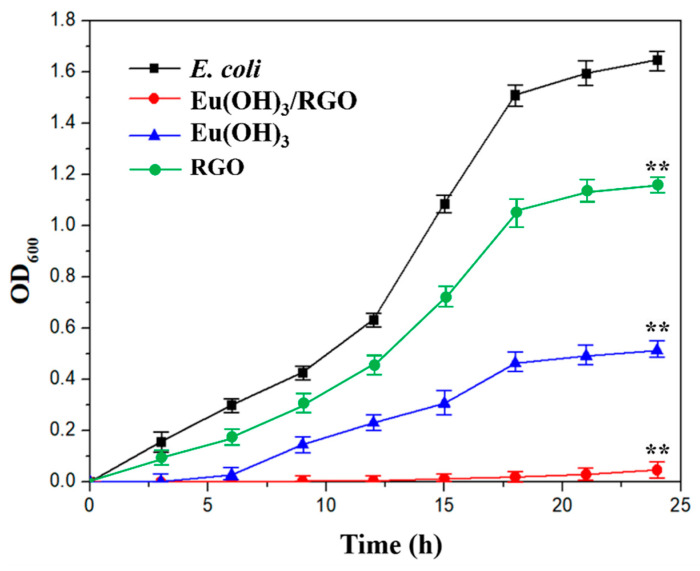
Growth curves of 100 mL *E. coli* suspension (10^4^ CFU/mL) exposed to 0.8 mg (8 µg/mL) of Eu(OH)_3_/RGO, Eu(OH)_3_, and RGO after 3, 6, 9, 12, 15, 18, 21, and 24 h. Error bars represent the standard deviations (n = 3; n = numbers of experimental results); ** *p* < 0.01 indicates growth curve at 24 h was statistically significant compared with the control group.

Figure 7 shows the relationship between the O.D. (OD600) of *E. coli* measured using the shaking flask method and culture time. For the analysis, a bacterial suspension (100 mL) was prepared and immersed in 1, 4, 8, 16, and 32 µg/mL of Eu(OH)_3_/RGO 220, which were tested to figure out the MIC against *E. coli*. With a decrease in the number of microbial cells, the OD value of the suspension decreased and the antibacterial activity increased. The antibacterial activity of the materials was measured at an interval of 3 h for a period of 24 h. The antibacterial activity of Eu(OH)_3_/RGO 220 increased with increasing its concentration. Moreover, the antibacterial activity depended on contact time and concentration of the sample. By increasing the concentration of the sample, the inhibition rate of bacteria increased. This result further confirmed that the MIC of Eu (OH) 3/RGO 220 was 8 µg/mL. Figure 8 shows the O.D. (OD600) of *E. coli* measured of 8µg/mL Eu(OH)_3_/RGO, Eu(OH)_3_, and RGO. The numbers of bacterial in the control group increased rapidly with time and decreased after 18 hours of culture. Furthermore, the growth curve for *E. coli* revealed that the O.D. of the Eu(OH)_3_/RGO 220, Eu(OH)_3_, and RGO samples decreased with time, indicating the effective antibacterial ability of the samples and the reduction in the growth rate. The growth curve showed significant differences in all groups at 24 h compared with the control group. These findings suggest that the Eu(OH)_3_/RGO 220 nanocomposites exhibited a stronger inhibition towards the growth of *E. coli*. This could be attributed to the addition of Eu^3+^ and the sharp edge of RGO, which not only effectively destroyed the protein structure and removed the free sulfhydryl groups(-SH) but also cut off the cell membrane and induced a loss in the important functions of the bacteria. Lastly, *E. coli* was cultured for 96 h, and the antibacterial activity of the samples was determined. The antibacterial activities of the materials were arranged in order of Eu(OH)_3_/RGO > Eu(OH)_3_ > RGO > Control.

### 3.4. Colony Counting Method

**Figure 9:**
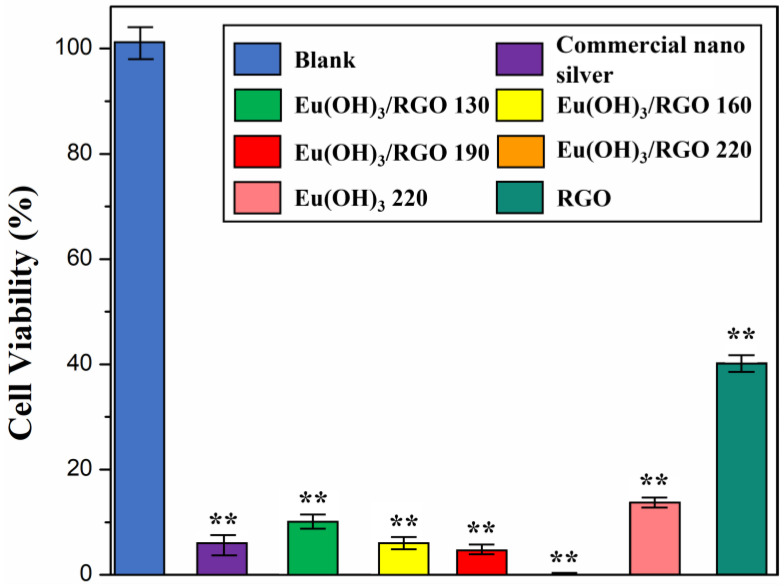
Viability rate (%) of different samples against *E. coli* (10^4^ CFU/mL) after 30 min. A concentration of 8 µg/mL was used for all the samples. Error bars represent the standard deviations (n = 3; n = numbers of experimental results); ** *p* < 0.01 indicates statistically significant compared with the control group.

The antibacterial properties of Eu(OH)_3_/RGO nanocomposite were quantitatively evaluated using the colony-forming counting method. The MIC investigation revealed that the MIC of the Eu(OH)_3_/RGO nanocomposite was 8 µg/mL. Therefore, this concentration was used for the colony counting test. The number of bacteria colonies observed in the culture treated with Eu(OH)_3_/RGO nanocomposite was significantly smaller than that of blank colonies, indicating that Eu(OH)_3_/RGO nanocomposite inhibited bacterial growth and effectively killed bacteria (Figure 9). Furthermore, after 30 min of culture, the viability rate of *E. coli* in the commercial nano silver, Eu(OH)_3_/RGO 130, Eu(OH)_3_/RGO 160, Eu(OH)_3_/RGO 190, Eu(OH)_3_/RGO 220, Eu(OH)_3_ 220, and RGO samples decreased to 7.0 ± 4.5%, 10.8 ± 3.1%, 7.2 ± 2.5%, 6.7 ± 2.0%, 0%, 17.3 ± 2.8%. and 40.0 ± 3.0% (n = 3, n = numbers of experimental results), respectively. The results indicated that there was a significant difference in all groups compared with the control group. In addition, the antimicrobial properties of the nanocomposites gradually increased with an increase in the synthesis temperature. This is because the particle size decreased and the crystal form became more compact with an increase in the synthesis temperature. Consequently, the small crystallite size and larger surface area of the nanocomposite significantly enhanced the chance of contact with bacteria. These results are consistent with the SEM and XRD results. Furthermore, the Eu(OH)_3_/RGO 190 and Eu(OH)_3_/RGO 220 nanocomposites reduced the viability of *E.coli* to levels below that of commercial nano silver (Figure 9). These results demonstrate the excellent antibacterial ability of the as-synthesized Eu(OH)_3_/RGO nanocomposites.

### 3.5. Inhibition Zone

**Figure 10:**
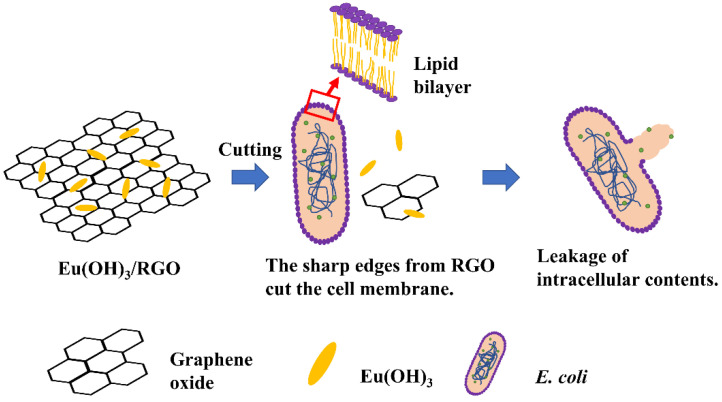
Plausible antibacterial mechanism of Eu(OH)_3_/RGO nanocomposites.

Multidrug-resistant *E. coli* are currently most difficult to treat with conventional antibiotics [54]. Therefore, in this study, the antibacterial activity of different samples against Gram-negative *E. coli* was investigated using the agar well diffusion method, and the results revealed that the antibacterial activity was comparable to that of the standard antibiotic Ampicillin. The MIC of RGO and Eu(OH)_3_ was greater than that of Eu(OH)_3_/RGO. To more clearly compare the zone of inhibition of *E. coli* suspensions by Ampicillin, RGO, Eu(OH)_3_, and Eu(OH)_3_/RGO, the sample amount was increased to 0.1 mg/mL. The inhibition zones of *E. coli* produced by 0.1 mg/mL of Ampicillin, RGO, Eu(OH)_3,_ and Eu(OH)_3_/RGO suspensions are presented in Table 3. The variation in the diameter of the inhibition zone reflects the antibacterial potency of the materials tested. A larger inhibition zone corresponds to an increased susceptibility of *E. Coli* to the antibacterial materials, whereas a smaller inhibition zone corresponds to a relatively low susceptibility to antibacterial materials. In this study, Eu(OH)_3_/RGO suspension exhibited the highest *E. coli* growth inhibition (21 ± 1.8 mm, n = 3, n = numbers of experimental results), followed by Ampicillin (18 ± 1.1 mm, n = 3), Eu(OH)_3_ (15 ± 1.1 mm, n = 3), and RGO (8 ± 1.7 mm, n = 3) suspension. The results indicate that there was a significant difference in all groups compared with a negative control. Eu(OH)_3_/RGO 220 exhibited a significantly higher activity, measured as the diameter of the inhibition zone, compared to those treated with RGO and Eu(OH)_3_. This could be attributed to the uniform loading of Eu(OH)_3_ nanorods on RGO, the extremely small particle size of Eu(OH)_3_ nanorods, and the synergistic effect between Eu(OH)_3_ and RGO. Generally, a smaller particle size with a higher surface area results in a higher antibacterial activity [55]. Figure 10 shows the plausible antibacterial mechanism of Eu(OH)_3_/RGO nanocomposites. The high antibacterial activity of the Eu(OH)_3_/RGO nanocomposites could be mainly attributed to the release of Eu^3+^ ions and the cutting of the cell membrane composed of a lipid bilayer by the sharp edge of RGO [56,57]. In addition, RGO wrapped the bacteria and prevented the entry of nutrients into the medium, thus leading to apoptosis [23]. The antibacterial activity of Eu(OH)_3_ nanorods depends on various mechanisms, such as size and electrostatic attraction between nanorods and the cell membrane of *E. coli* and the liberation of Eu^3+^ ions [57,58]. In addition, the infiltration of the released Eu^3+^ ions into the bacterial cells and the electrostatic interactions between bacteria cell walls and nanorods can result in the death of the bacteria [59,60]. Particularly, the Eu(OH)_3_/RGO nanocomposites retained the antibacterial activity of RGO, which was further enhanced by the presence of Eu(OH)_3_ nanorods in the nanocomposite.

### 3.6. Reusability of Eu(OH)_3_/RGO Nanocomposites

**Figure 11:**
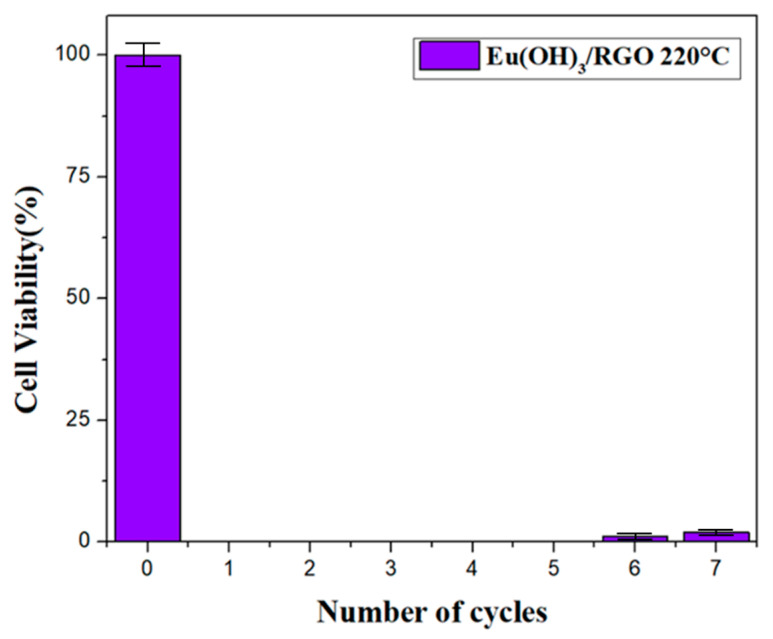
Reusability of 8 µg/mL Eu(OH)_3_ nanocomposites against 100 mL of *E. coli* (10^4^ CFU/mL) at 37 °C and 120 RPM for 30 min. Error bars represent the standard deviations, n = 3, n= numbers of experimental results.

To verify the cyclic antibacterial performance of the as-synthesized nanocomposite, Eu(OH)_3_/RGO 220 was collected after every measurement for seven recycling times. The above reported results indicated that Eu(OH)3/RGO 220 was the most effective of the materials tested. Therefore, the Eu(OH)_3_/RGO 220 sample was used for the recycling test. Figure 11 shows the results of the Eu(OH)_3_/RGO usability tests against *E. coli* colonies. The results revealed an *E. coli* viability of 0% was maintained and no colonies were formed after the fifth recycling of Eu(OH)_3_/RGO. In addition, after the seventh recycling, the *E. coli* viability did not exceed 2%, indicating the reusability and stability of the Eu(OH)_3_/RGO nanocomposite.

## 4. Conclusions

In this study, Eu(OH)_3_/ RGO nanocomposites were successfully synthesized and characterized using a microwave-assisted, one-step method. The effects of reaction temperature on the morphology and antibacterial activity of the nanocomposites were examined. SEM analysis revealed that the synthesized nanocomposite exhibited a uniform distribution of Eu(OH)_3_ on the RGO sheet. In addition, the SEM images revealed that the particle size gradually decreased with increasing synthesis temperature. Furthermore, XRD analysis revealed the presence of peaks corresponding to the standard Eu(OH)_3_, which confirmed the crystal structure of nanocomposite materials. In addition, the Raman spectra, FT-IR spectra, photoluminescence spectra, and SEM surface morphology images confirmed the successful synthesis of the Eu(OH)_3_/RGO nanocomposite. The antibacterial ability of the nanocomposites on *E. coli* was investigated in an aseptic laboratory, and the results revealed that the Eu(OH)_3_/RGO nanocomposites exhibited superior antibacterial activities compared to Eu(OH)_3_ and RGO alone. Furthermore, RGO had a significant effect on the dispersion of Eu(OH)_3_ nanoparticles on its surface and the prevention of metal nanoparticle aggregation. The reusability results revealed that the Eu(OH)_3_/RGO 220 nanocomposite exhibited excellent antibacterial activity and stability. The Eu(OH)_3_/RGO 220 nanocomposite synthesized in this study is an efficient, fast, and reusable antimicrobial agent. These results indicate the promising potential of this nanocomposite as an antimicrobial agent.

## Figures and Tables

**Table 1:** Average rod lengths (±standard deviation, SD) of Eu(OH)_3_, Eu(OH)_3_/RGO 220, Eu(OH)_3_/RGO 190, Eu(OH)_3_/RGO 160, and Eu(OH)_3_/RGO 130.

Sample	Rod Length (nm)
Eu(OH)_3_ 220	600 ± 9.8
Eu(OH)_3_/RGO 130	630 ± 9.5
Eu(OH)_3_/RGO 160	471 ± 7.2
Eu(OH)_3_/RGO 190	158 ± 2.2
Eu(OH)_3_/RGO 220	97 ± 2.4

Each value is expressed as mean ± SD (n = 3).

**Table 2:** Minimum inhibitory concentrations (MIC) of RGO, Eu(OH)_3_, and Eu(OH)_3_/RGO.

Bacterial Strain	MIC (µg/mL)		
	RGO	Eu(OH)_3_	Eu(OH)_3_/RGO
*E. coli*	256	32	8

**Table 3:** Diameter (± standard deviation, SD) of 150 µL of the *E. coli* (10^4^ CFU/mL) inhibitory zone.

	Zone of Inhibition (Diameter ± SD (mm))			
	Ampicillin	RGO	Eu(OH)_3_	Eu(OH)_3_/RGO
*E. coli*	18.3 ± 1.1 **	8.1 ± 1.7 **	15.0 ± 1.1 **	21.2 ± 1.8 **

Each value is expressed as mean ± SD (n = 3); ** *p* < 0.01 indicates statistically significant compared with negative control.

## Data Availability

All data contained within the article.
